# Using the Personalized Advantage Index for Individual Treatment Allocation to Blended Treatment or Treatment as Usual for Depression in Secondary Care

**DOI:** 10.3390/jcm9020490

**Published:** 2020-02-11

**Authors:** Nadine Friedl, Tobias Krieger, Karine Chevreul, Jean Baptiste Hazo, Jérôme Holtzmann, Mark Hoogendoorn, Annet Kleiboer, Kim Mathiasen, Antoine Urech, Heleen Riper, Thomas Berger

**Affiliations:** 1 Department of Clinical Psychology, University of Bern, 3012 Bern, Switzerland; 2 URC Eco Ile-de-France (AP-HP), Hotel Dieu, 1, Place du Parvis Notre Dame, 75004 Paris, France; 3 Eceve, Unit 1123, Inserm, University of Paris, Health Economics Research Unit, Assistance Publique-Hôpitaux de Paris, 75004 Paris, France; 4 University Hospital Grenoble Alpes, Mood Disorders and Emotional Pathologies Unit, Pôle de Psychiatrie, Neurologie et Rééducation Neurologique, 38043 Grenoble, France; 5 Department of Computer Science, VU University Amsterdam Faculty of Sciences, De Boelelaan 1081m, 1081 HV Amsterdam, The Netherlands; 6 Section Clinical Psychology, Faculty of Behavioural and Movement Sciences, Vrije Universiteit Amsterdam and EMGO+ Institute for Health Care and Research, Van der Boechorststraat 1, 1081 BT Amsterdam, The Netherlands; 7 Department of Psychology, Faculty of Health Sciences, University of Southern Denmark, 5230 Odense M, Denmark; 8 Center of Telepsychiatry, University of Southern Denmark, 5000 Odense, Denmark; 9 INSELSPITAL, University Hospital Bern, University Clinic for Neurology, University Acute-Neurorehabilitation Center, 3010 Bern, Switzerland; 10 Department of Psychiatry and the Amsterdam Public Health Research Institute, GGZ inGeest/Amsterdam UMC, Vrije Universiteit, de Boelelaan 1117, 1081 HV Amsterdam, The Netherlands; 11 Department of Research and Innovation, GGZ inGeest Specialized Mental Health Care, Oldenaller 1, 1081 HJ Amsterdam, The Netherlands; 12 Department of Clinical, Neuro-and Developmental Psychology and the Amsterdam Public Health Research Institute, Vrije Universiteit Amsterdam, van der Boechorststraat 7, 1081 BT Amsterdam, The Netherlands

**Keywords:** personalized advantage index, depression, blended treatment, CBT, treatment selection, Bayesian model averaging

## Abstract

A variety of effective psychotherapies for depression are available, but patients who suffer from depression vary in their treatment response. Combining face-to-face therapies with internet-based elements in the sense of blended treatment is a new approach to treatment for depression. The goal of this study was to answer the following research questions: (1) What are the most important predictors determining optimal treatment allocation to treatment as usual or blended treatment? and (2) Would model-determined treatment allocation using this predictive information and the personalized advantage index (PAI)-approach result in better treatment outcomes? Bayesian model averaging (BMA) was applied to the data of a randomized controlled trial (RCT) comparing the efficacy of treatment as usual and blended treatment in depressive outpatients. Pre-treatment symptomatology and treatment expectancy predicted outcomes irrespective of treatment condition, whereas different prescriptive predictors were found. A PAI of 2.33 PHQ-9 points was found, meaning that patients who would have received the treatment that is optimal for them would have had a post-treatment PHQ-9 score that is two points lower than if they had received the treatment that is suboptimal for them. For 29% of the sample, the PAI was five or greater, which means that a substantial difference between the two treatments was predicted. The use of the PAI approach for clinical practice must be further confirmed in prospective research; the current study supports the identification of specific interventions favorable for specific patients.

## 1. Introduction

Globally, 300 million people of all ages suffer from depression [1]. Depression is one of the most common problems seen in clinical practice, and it is associated with high societal costs, as well as great suffering [2]. Given this burden, the need for improved access to efficacious and cost-effective treatments is essential [3]. In the last decades, research has focused on examining different treatment options for depression. Especially, cognitive behavior therapy (CBT) and interpersonal therapy (IPT) can be seen as first-line treatments [4,5]. Moreover, current studies are aimed at scaling up treatments for depression. One way to do so is through internet-based therapies [5]. Whereas the most dominant format in which treatment is delivered is through face-to-face contact, internet-based therapies have received much attention in recent years [6]. The efficacy and cost-effectiveness of the latter have been supported by a growing number of research [7,8,9]. Even though only a few studies have directly compared internet-based with face-to-face CBT for depression, results suggest it to have similar overall effects [6].

A newer approach to depression treatment is to combine web-based technologies with face-to-face therapy, called blended treatment. Blended treatment includes any combination of face-to-face therapy and internet-based interventions, e.g., web-based components are used as an adjunctive intervention or are integrated during face-to-face therapy [10]. Although research that investigates the efficacy of blended treatment formats is still scarce, preliminary results suggest their feasibility and their efficacy in reducing symptoms of depression [11,12,13,14,15]. For example, a randomized controlled trial by Berger and colleagues [16] showed the superiority of blended treatment, consisting of an internet-based intervention as an adjunct to face-to-face psychotherapy, in comparison to regular face-to-face psychotherapy in a pragmatic randomized controlled study in patients with a unipolar affective disorder in routine care. Another recent study showed the noninferiority of blended treatment to conventional CBT for patients with depression and found blended treatment to be cost-effective [17]. Moreover, the blended treatment has also been evaluated in an inpatient setting where patients suffering from depression that received an online self-help program in addition to inpatient psychotherapy improved significantly more than patients who received online information about depression in addition to inpatient psychotherapy [18]. Furthermore, a recent systematic review showed that, compared to face-to-face therapy, a blended treatment may help maintaining initially achieved changes within psychotherapy in the long-term [19].

Potential benefits of blending treatments may be a greater reduction in depressive symptoms and increased cost-effectiveness [20], as well as an improvement of patients’ adherence to the treatment program [21]. Furthermore, an asset of a blended treatment may be that it combines the advantages of both treatment forms [3,14,22,23]. For example, the face-to-face contact enables clinicians to individualize or tailor the treatment and to react in crisis situations, while providing online modules between sessions could promote patient engagement and enhance the translation of treatment into daily life (e.g., [24]). On the other hand, when online components are not used by the patients in blended treatments, reductions in the number of face-to-face sessions may lead to worse treatment outcomes [25]. Furthermore, therapists may raise concerns of overburdening depressed patients [18]. So far, it is not clear for which patients blended treatment may be a feasible option and for which patients a conventional treatment should be favored.

Patients with depression may differ substantially from each other, and evidence suggests that the diagnostic categories leave room for great diversity [26,27]. This results in differences with regard to patients’ illness courses and individual treatment responses [28]. Research suggests that individual patient characteristics may moderate the efficacy of different treatments at an individual level [29]. It is, therefore, important to recognize that no single treatment is likely to be the best for everyone, even though, on a group level, it is efficacious for patients suffering from depression [26,30]. This is why more and more researchers move away from investigating treatment efficacy on a group level and instead focus on custom-tailoring the treatment to the individual patient [30,31]. In this sense, it may be a solution to increase the overall treatment response rates [32].

Precision medicine tries to tailor treatments to the specific needs of the patient [33]. More recently, in clinical psychology and psychotherapy, algorithms are used that predict from which treatment a patient benefits the most [34]. As an example, Becker and colleagues [35] introduce a conceptual framework that helps classifying applications of predictive modeling in mental health research. These authors try to bridge the gap between psychologists and predictive modelers with providing a common language for classifying predictive modeling mental health research. They suggest that e-mental health researchers should focus more on the validity of model predictions instead of solely focusing on identifying predictors. Another example is the personalized advantage index PAI) approach, which identifies patients with a certain disorder (e.g., major depression) who benefit more from one treatment than another [30]. Using the personalized advantage index (PAI), it is possible to identify the treatment that predicts a better treatment outcome for a given patient if there are two comparably effective treatments to choose from [36]. The PAI estimates how much a specific treatment is better for an individual patient than another, and its feasibility and relevance have been shown in several studies on the treatment of depression [36,37,38,39,40]. Baseline patient characteristics can be divided into two types of predictors: a prognostic variable predicts treatment outcome irrespective of treatment condition, whereas a prescriptive variable predicts a differential treatment response to two or more treatment modalities [29,30]. Up to today, different treatments have been compared using the PAI, and its values range from 1.4 when comparing CBT to CBT with integrated exposure and emotion-focused elements [38] up to 8.9 when comparing cognitive therapy to IPT [40]. Higher absolute values of the PAI stand for stronger predicted benefits of one treatment over another. Being able to identify the best treatment for an individual with depression is essential, because it may make health care delivery more efficient [32]. Even though predictive modeling is still very young in the field of e-mental health [41], Bremer and colleagues [42] were able to predict clinical outcomes and costs of patients with depression prior to starting blended psychotherapy in a subsample of the current study using machine learning techniques.

In the current study, treatment as usual (TAU), i.e., regular face-to-face psychotherapy, was compared to blended treatment for patients with major depressive disorder (MDD) in secondary care. The present study set out to answer the following research questions: (1) What are the most important predictors determining optimal treatment allocation to TAU or blended treatment? and (2) Would model-determined treatment allocation using this predictive information and the PAI-approach result in better treatment outcomes? To the best of our knowledge, this is the first study comparing different treatment delivering formats, i.e., traditional face-to-face CBT versus blended CBT, by using the PAI-approach.

## 2. Materials and Methods

Data used in the present study was drawn from the European project “European COMPARative effectiveness research on blended depression treatment” (E-COMPARED, February 2018) [43]. The E-COMPARED project included a randomized, controlled, noninferiority trial that examined the clinical and cost-effectiveness of blended treatment compared to treatment as usual in routine care in nine European countries. Adult patients diagnosed with MDD were recruited in primary or in specialized mental health care. The current study uses the data of the four countries that recruited patients in specialized mental health care (France, the Netherlands, Switzerland, and Denmark). The following inclusion criteria were met by participants: (1) being 18 years of age or older, (2) meeting Diagnostic and Statistical Manual of Mental Disorders (DSM-IV) diagnostic criteria for MDD as confirmed by a telephone-administered MINI international neuropsychiatric interview (M.I.N.I.) version 5.0 [44], and (3) minimal to severe symptoms of depression based on a score of 5 or above on the patient health questionnaire-9 (PHQ-9) [45]. Exclusion criteria for participating in the study were: (1) high risk for suicide according to the M.I.N.I.; (2) psychiatric comorbidity such as substance dependence, bipolar affective disorder, psychotic illness, or obsessive compulsive disorder; (3) currently receiving another psychological treatment for depression; (4) being unable to comprehend the spoken and written language of the country where the study is conducted; (5) not having access to a computer with fast internet connection; and (6) not having a smartphone that is compatible with the mobile component of the intervention that is offered. Patients were randomized to blended treatment or TAU using an allocation scheme with a computerized random number generator at an allocation ratio of 1:1 and between 8 and 14 allocations per block. Details about the study design are described elsewhere [43]. For Switzerland, this study was approved by the cantonal ethics committees Bern and Zurich (registration number: 001/15; date: 18 March 2015); for Denmark, the study was approved by the Ethics Committee of the Region of Southern Denmark (registration number S-20150150; date: 18 November 2015); for France, the study was approved by the “Comité de protection des personnes”, Ile de France V (registration number 15033-*n*° 2015-A00565-44; date: 2 June 2015); and, for the Netherlands, the study was approved by the METC VUMC (registration number 2015.078; date: 8 May 2015). Furthermore, all participants provided written informed consent and gave permission to all E-COMPARED partners to use their anonymized data.

### 2.1. Sample

The current study has a sample size of *n* = 251. The sample consists of 83 participants from the Netherlands (33.9%), 79 participants from France (32.2%), 44 from Switzerland (18.0%), and 39 participants from Denmark (15.9%). The mean age at baseline was 41.0 years (SD = 13.7), and 68.2% of the participants were female. The majority were either single (33.5%) or married (31.8%), and 21.2% of participants were living together and 12.8% were divorced. In the TAU condition, 57.9% of the sample suffered from a recurrent depression, 45.2% from a current melancholic depressive episode, 7.9% from a comorbid dysthymia, and 46.8% from a comorbid anxiety disorder. The number of patients are taking antidepressant medication at the time of the baseline measurement was 53.2%. In the blended treatment condition, 53.8% of the participants suffered from a recurrent depression, 34.5% from a current melancholic depressive episode, 5.9% from a comorbid dysthymia, and 61.3% had a comorbid anxiety disorder. Half of the participants (49.6%) were taking antidepressant medication at baseline.

### 2.2. Interventions

Individual face-to-face CBT was combined with internet-based CBT elements delivered through a platform for blended treatment. Three different online platforms were used across the participating countries. Switzerland used “Deprexis” [46], whereas Denmark used NoDep [47] and France and the Netherlands used the platform “Moodbuster” [3]. The most important components of the treatment were cognitive restructuring, behavioral activation, psychoeducation, and relapse prevention, which were delivered over 11–20 sessions. In the blended treatment, a smaller number of face-to-face sessions is offered, and some sessions are replaced by online modules. Treatment was provided by CBT therapists who received special training on how to deliver the blended treatment. In Switzerland and the Netherlands, the therapists were either licensed CBT therapists or CBT therapists who were supervised by an experienced licensed CBT therapist. In Denmark, the treatment was delivered by either licensed psychologists or psychologists under supervision of licensed psychologists. In France, the blended treatment was provided by licensed psychotherapists [43].

The TAU treatment was defined as the routine care that patients received in specialized mental health care when they were diagnosed with depression. In practice, this meant that the TAU group received regular fact-to-face CBT.

### 2.3. Measures

#### 2.3.1. Primary Outcome

The primary outcome measure for this study was the PHQ-9 [45] assessed after 12 weeks. The PHQ-9 consists of nine questions which are based upon the DSM-IV criteria for the diagnosis of depressive disorders. It is used as a diagnostic instrument and as a severity measure for depression. A 5-point difference in PHQ-9 scores is seen as clinically significant [48]. The validity and sensitivity to change of the PHQ-9 were satisfactory in previous studies [49,50]. Cronbach’s alpha in the present study was 0.78.

#### 2.3.2. Predictor Variables

We used an exploratory approach and included a total of 28 potential predictors measured at baseline in the analysis. All variables of the baseline assessment that did not exceed a number of acceptable missing values (<50%) were included. We classified the variables into four categories: (1) sociodemographic variables, (2) symptomatology and quality of life, (3) healthcare utilization, and (4) patient expectancy.

The sociodemographic variables included age, marital status, education, gender, and country and were assessed with single item questions. Variables related to symptomatology and treatment history were recurrent depression, therapy preference, dysthymia, melancholic depressive episodes, comorbid anxiety, and current use of antidepressants. Quality of life was measured with the EQ-5D [51]. Healthcare utilization was assessed with the TiC-P [52]. The TiC-P examines healthcare consumption and productivity losses as a consequence of a mental disorder via a self-report questionnaire. The questions include contacts within the healthcare sector and the use of medication. All the questions aim at the period of the last four months before the start of the treatment (see Table 1 for the items of the TiC-P that have been included). Patient expectancy was measured by the credibility and expectancy questionnaire (CEQ; [53]).

### 2.4. Data Analytical Strategy

Regarding the predictor variables, a bottom-up approach was followed, which means that even though some variables might have a particular relevance to one treatment or the other, the predictors are treated equally in the data analysis. 

#### 2.4.1. Missing Data

In line with previous research using the PAI approach, we included those participants for which PHQ-9 scores after 12 weeks were available [36,38,40]. This left us with *n* = 245, representing 97.6% of the total sample. Distributed over the two conditions, there were 126 patients in the TAU condition and 119 patients in the blended treatment condition. With regard to the baseline measures, missing values were found in the dimensions credibility and expectancy of the CEQ (3.7% respectively 4.1%), the EQ-5D (1.6%), antidepressants (1.2%), prior psychotherapy (40.4%), comorbid melancholic episodes (14.3%), comorbid dysthymia (5.3%), comorbid anxiety (2.4%), and some items of the TiC-P (2.0–40.8%). We imputed these missings in the baseline measures with the R *missForest* [54]. Here, missing values are predicted on the basis of a random forest approach, trained on observed values of the available data. An advantage of *missForest* is that it imputes categorical and continuous variables simultaneously [55]. It has been shown to be highly accurate and to outperform other imputation methods due to its small imputation error [56]. The imputed data set is the basis for all analyses that follow.

#### 2.4.2. Bayesian Model Averaging (BMA)

There are different data analysis approaches that can be used to identify baseline variables that predict outcome in one treatment versus another. Data analysis approaches that rely on one model only can only include a limited number of baseline measures that could be predictors. In that case, the most common rule that is used states that at least 10 observations per predictor are necessary to not exceed a level of bias which is acceptable [57,58]. If we had followed this approach, we could have only included a small number of predictors. Furthermore, the problem with relying upon a single selected model is that it may result in overconfidence in the conclusions drawn regarding quantified associations. The problem is that there may be alternative models that have different subsets of predictors that fit the data just as well as the one selected model [59,60]. Overfitted models cause uncertainty regarding the actual value of findings, because they may not replicate in future samples [61]. Bayesian model averaging (BMA) is a method that can account for model uncertainty while providing a better predictive ability. The BMA method has two advantages. First, it results in predictions that are less risky and. second, BMA provides simpler model choice criteria, because it uses the Bayesian inference for model prediction and selection [62]. With BMA, a posterior probability is estimated on the basis that each considered model is the correct one. This included the aforementioned model uncertainty in the estimates for the parameter and inferences. That means that BMA averages over all possible predictor sets and delivers model choice criteria that help to identify the most probable model. With using BMA, sharper predictions can be derived from the data, especially in cases with many possible predictors but a limited sample size. Several studies have supported BMA’s predictive performance [60,63,64,65,66].

The data frame was divided into two subsets: the TAU condition and the blended treatment condition. Using the R package *BAS*, for each condition, a separate linear regression model was computed [67]. Then, Bayesian adaptive sampling (BAS) without replacement for variable selection in linear models using the function *bas.lm* with treatment outcome was applied (PHQ-9 score after 12 weeks) as the dependent variable. The relative importance of each variable was evaluated using the posterior probabilities that were calculated for each potential predictor. The marginal posterior inclusion probabilities functioned as the criteria for determining the importance of the potential predictors. Values above 0.5 point out that the predictor has been incorporated in more than half the models; thus, in the present study, in over 15,000 models. The nominal variables were split into their different categories, which enabled a precise interpretation of the results. The model performance was further evaluated using posterior probabilities. The appropriate *bas.lm* function was chosen based on the following considerations: a Laplace approximation to the Jeffreys-Zellner-Siow (JZS) prior for the integration of alpha = 1 was used as the criterion for the priors, which is called the “ZS-null”. The JZS prior uses the Zellner-Siow Cauchy prior on the coefficients and the Jeffreys prior on sigma. The squared scale of the prior, where the default is alpha = 1, can be controlled using the optional parameter “alpha” [38,67]. Marginal inclusion probabilities were calculated with the “MCMC+BAS” method, which runs an initial Markov chain Monte Carlo (MCMC) algorithm and then samples without replacement, as in BAS. Compared to the BAS alone, the “MCMC+BAS” method is the preferred option, because it provides estimates with low bias [68]. The number of models was set to 30,000 assuming that each additional model would add only a small increment to the cumulative probability, i.e., not leading to essential differences in posterior distributions. Due to the limited sample size, the models have been built and tested in the same dataset.

#### 2.4.3. Personalized Advantage Index (PAI)

When predicting the therapy outcome for each patient, applying a leave-one-out approach (“jackknife”) to estimate regression models is an essential beginning step in generating the PAI [36,38,69]. In this procedure, overfitting can be avoided by excluding each target patient for whom the PAI prediction is estimated from the model. For each patient, two regression models were built using the treatment-specific predictors identified with BMA. For each patient, a factual prediction (PHQ-9 score at 12 weeks of the treatment the patient has received) and a counterfactual prediction (PHQ-9 score at 12 weeks of the intervention the patient did not receive) were estimated. In the next step, those two predictions were compared, and the prediction that resulted in the best outcome for the patient was defined to be the optimal treatment for that patient. When comparing the observed change scores, the size of the predicted difference of receiving the treatment with the greater predicted benefit is ultimately the PAI [36]. The higher the absolute values of the PAI, the stronger is the predicted benefit of one treatment over another. The interpretation of the PAI can be demonstrated with a recent study that used the PHQ-9 as the primary outcome and found a PAI of 2.5 [70]. This means that if patients had received their “optimal” treatment (out of the two), their PHQ-9 score at 12 weeks would have been 2.5 points lower than if they had obtained their nonoptimal treatment.

## 3. Results

Henceforth, we firstly report the five best models for each treatment condition, and, secondly, we report the PAI results. The best models are defined based on the highest posterior probability and the lowest Bayesian information criterion (BIC). 

### 3.1. Variables Predicting Outcome in TAU

The five best models predicting depression severity at 12 weeks in the TAU condition are displayed in Table 2. The Bayes factor, number of predictors, R^2^, log marginal likelihood, and the posterior probabilities are provided for each model. Model 1 has the largest Bayes factor and the largest posterior probability (0.02) and, thus, seems to fit the data best. As a result, Model 1 was selected as our final predictive model of the PHQ-9 score at 12 weeks in the TAU condition.

While the selected model includes six variables in total, the strongest predictors of the PHQ-9 score at 12 weeks in the TAU condition included the pretreatment PHQ-9 score (Prob = 100%), CEQ expectancy (Prob = 97%), “How many days did you use outpatient psychotherapeutic services in addition to your psychotherapy?” (Prob = 95%), “How many times did you consult a psychiatrist?” (Prob = 64%), Denmark (Prob = 58%), and “How many days did you attend a day-time treatment program in a psychiatric hospital?” (Prob = 51%). A higher pretreatment score, more consultations with a psychiatrist, and more days in a day-time treatment program in a psychiatric hospital prior to treatment predicted a higher PHQ-9 score at 12 weeks. Higher expectancy scores, receiving TAU in Denmark, and more days using outpatient psychotherapeutic services in addition to the psychotherapy prior to treatment predicted lower PHQ-9 scores at 12 weeks. The effects of other variables appeared minimal due to their small posterior probabilities. See Appendix 1 in the Supplemental Online Material for the complete list of variables and their inclusion probabilities.

### 3.2. Variables Predicting Outcome in the Nlended Treatment

Table 3 gives an overview of the five best models to predict treatment outcome in the blended treatment condition. Based on the posterior probabilities and the Bayes factor, Model 1 was rated the best model. Thus, Model 1 was selected as the final predictive model for the blended treatment condition.

Based on the posterior probabilities, the most important predictors for treatment outcome in the blended treatment were the pretreatment PHQ-9 score (Prob = 99.9%), regular hospital admissions (Prob = 99.9%), EQ-5D quality of life (Prob = 74.6%), CEQ expectancy (Prob = 72.3%), consulting self-help groups (Prob = 70.0%), and being widowed (Prob = 49.7%). CEQ credibility reached a posterior probability of 42.9%. A higher pretreatment PHQ-9 score, being widowed, more hospital admissions, and consulting self-help groups predicted higher PHQ-9 scores at 12 weeks. A higher expectancy for improvement and a higher quality of life predicted lower PHQ-9 scores after 12 weeks. See Appendix 2 in the Supplemental Online Material for the posterior probabilities of all variables measured at baseline.

### 3.3. Personalized Advantage Index

**Figure 1:**
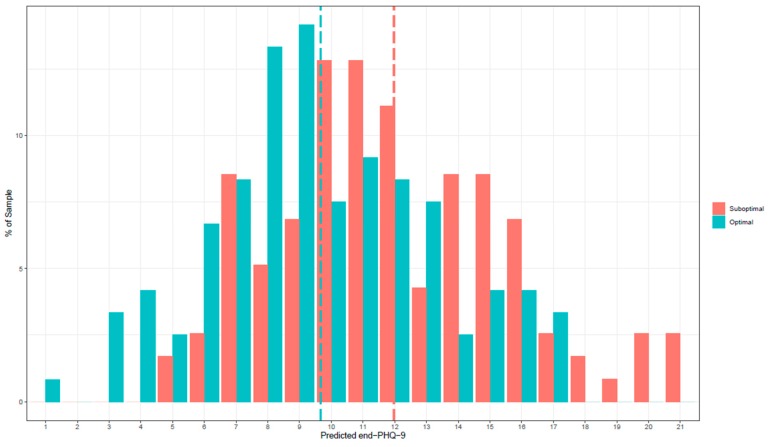
Frequency of predicted PHQ-9 scores at 12 weeks.

Using the treatment specific predictors described above, the prediction of a patient’s PHQ-9 score after 12 weeks was computed separately for each treatment condition. The true error of the PHQ-9 score predictions at 12 weeks was 4.16, representing the average absolute difference between the predicted and actual, observed scores across all patients. Patients who were categorized as having received their optimal treatment had a mean PHQ-9 score of 9.67 (*n* = 124) at 12 weeks, whereas patients who were classified as having received their suboptimal treatment had a mean PHQ-9 score of 12.00 (*n* = 121). Figure 1 shows the frequency of predicted PHQ-9 scores at 12 weeks for every patient in both the optimal and suboptimal treatments.

**Figure 2:**
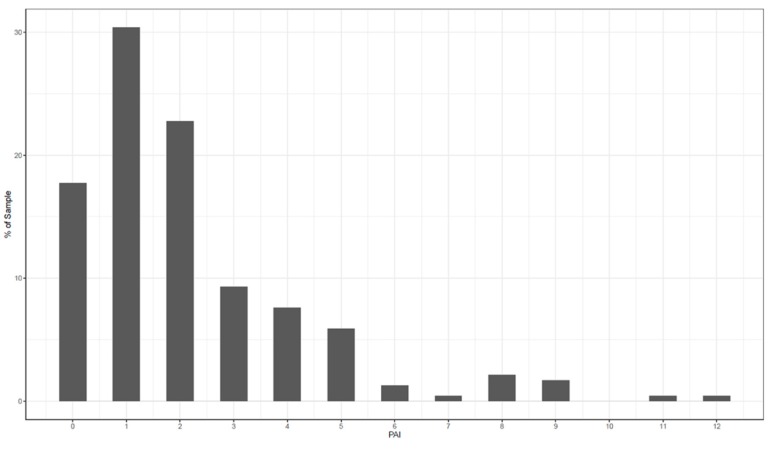
Frequencies of individual personalized advantage indexes (PAIs).

In the first step, an individual PAI was calculated for each patient. Secondly, the average PAI was calculated as the mean difference in PHQ-9 scores between the optimal and the suboptimal treatments for each patient. The average PAI of the current study was 2.33. The PAI can be read as follows: if patients had received the treatment that is “optimal” for them, their PHQ-9 score at 12 weeks would have been 2.33 points lower than if they had received the treatment that is suboptimal for them. In Figure 2, the frequencies of the individual PAIs are shown. A PAI that is five or greater would mean that a substantial difference was predicted between the two treatments, because 5 points on the PHQ-9 stands for a minimal clinically meaningful difference for individual change [71]. This was the case for 29% of the patients in this sample.

## 4. Discussion

Regarding the predictors of treatment outcome at 12 weeks in each of the interventions, different relevant predictors were identified for TAU and the blended treatment, respectively. In the TAU condition, a lower pretreatment PHQ-9 score, less consultations with a psychiatrist and less days in a day-time treatment program in a psychiatric hospital, higher expectancy, receiving TAU in Denmark, and more days using outpatient psychotherapeutic services in the four months prior to the study predicted a better treatment outcome, i.e., a lower PHQ-9 score (at 12 weeks). In contrast, in the blended treatment condition, a lower pretreatment PHQ-9 score, not being widowed, less hospital admissions and consulting self-help, a higher expectancy for improvement, and a higher EQ-5D score predicted lower scores at 12 weeks; thus, a better treatment outcome.

To offer an initial interpretation of our findings, the distinction between prescriptive and prognostic predictors is used. Prognostic variables predict treatment outcomes regardless of treatment conditions [30,38]. In contrast, prescriptive variables may support differential indications by predicting whether a patient will benefit more from one treatment in comparison to another. In the present study, the pretreatment depressive symptomatology and treatment expectancy are the only prognostic predictors, i.e., the only variables that predict treatment outcome in both conditions. This is in line with previous research that has found pretreatment symptomatology and expectancy to be important predictors of treatment outcome, in the sense that higher symptomatology before treatment predicts worse end-state symptomatology [30,34,36,38,72,73] and higher expectancy for improvement predicts better treatment outcome [74,75]. Interestingly, for internet-based treatments, higher baseline symptomatology is not necessarily a negative predictor of treatment outcome. More often the opposite is found, i.e., that higher depressive symptomatology pretreatment predicts better treatment outcomes [76,77,78]. This might be partly explained by the efficacy nature of previous randomized controlled trials (RCTs) in comparison to the routine care and effectiveness nature of the current study.

Regarding the prescriptive predictors, our findings partly corroborate findings from previous studies predicting treatment outcomes for patients with depression. With regard to prescriptive predictors of the blended treatment condition, a lower quality of life and being widowed predicted worse treatment outcomes. In contrast to the present result, the study by Huibers and colleagues [40] found a higher quality of life to be a prognostic predictor, i.e., to predict favorable outcomes irrespective of treatment conditions.

For the TAU condition, more consultations with a psychiatrist and more days in a day-time treatment program in a psychiatric hospital predicted worse treatment outcomes. This could mean that patients’ symptomatology and patients’ general functioning is too severe to be able to profit from TAU. Furthermore, to our knowledge, there are no international studies regarding psychotherapy of depression, indicating country as a relevant predictor of outcome.

Healthcare utilization within the four months prior starting treatment was found to be a prescriptive predictor in both conditions. Healthcare uptake may be a proxy for a higher somatic or mental burden and/or may represent a more severe symptomatology of depression. In previous studies, more complex cases (e.g., with chronic symptoms and psychiatric comorbidities) or more severe depressive symptomatology predicted a worse therapy outcome [29,38,79,80,81]. Interestingly, more hospital admissions only predicted worse treatment outcomes in the blended treatment condition. A possible interpretation of this finding is the fact that the online modules in the blended treatment protocol are highly standardized to target depressive symptomatology. As a result, they may have not sufficiently addressed comorbid symptoms. Somatic comorbidities in patients with depression are not a scarcity and have an influence on individual treatment response and illness course, because they can complicate treatment [81]. Furthermore, the number of hospital admissions may also be a proxy for case complexity and higher mental burden, which in turn may have a negative impact on treatment outcome.

This study’s results demonstrate that BMA makes it possible to use a limited set of baseline variables to predict treatment outcome. This is in line with a recent study by Bremer and colleagues [42] that showed the feasibility of providing personalized treatment recommendations at baseline regarding clinical and cost-effectiveness using a subsample of the current study by evaluating various machine learning techniques. Moreover, the present study showed that despite sharing the same diagnosis, patients might benefit more from different treatments. The current study found a PAI of 2.33, indicating that patients could have a PHQ-9 score at 12 weeks that is, on average, more than two points lower if they receive their model-determined optimal treatment in comparison to the suboptimal treatment. This value is in the range of other studies that have found PAIs ranging from 1.35 [38] up to 8.9 [40]. Importantly, for almost one-third (29%) of the patients in the present study, a substantial difference was predicted between the two treatment modalities as the individual PAI was 5 or greater. This result is in line with increasing evidence suggesting that differential treatment responses are not rare and might play an important role for an individual patient and the health care system [30].

The current study has several limitations. First of all, the relatively small sample size did not allow us to build and test the models in separate samples. Using the same sample for testing and building the model might lead to a potential risk of overconfidence. Nevertheless, if studies are designed to develop and validate prescriptive prediction scores that can be tested in future hypothesis-driven confirmatory studies, a smaller sample size might be legitimate [82]. Secondly, the results are based solely on self-reports, and future studies should also include observer ratings. Third, the restricted set of baseline measures is another limitation. Constructs such as personality traits or the familiarity with computers have not been assessed but may have influenced engagement with the online component. Relatedly, people with a low socioeconomic status or senior citizens may not have been well represented in the present study sample. Such groups may not have the opportunity to benefit from the blended treatment, because they may not have access to a computer or a smartphone and/or lack the knowledge to use them. As a consequence, the restricted sample in the present study limits the generalizability of the results. Furthermore, the current study predicts treatment outcome after 12 weeks. Future studies should predict long-term treatment outcome [83]. Finally, we have followed a data-driven approach instead of a theory-driven approach. Although the two methods should be considered complementary [84], a disadvantage of data-driven research is that it is not experiential, and relying on the data alone might not capture the whole picture. Relatedly, the predictors found in the current study need to be validated and replicated in future hypothesis-driven studies.

In spite of the limitations above, the current study is promising to contribute to the further understanding of treatment for depression, because it investigates implications for the use of the blended treatment for patients with depression. Clinical practice should consider factors found to play a role in the treatment and processes of change to provide the optimal treatment for each individual. For example, the quality of life should be taken into account, as these patients may need a more intense treatment protocol that integrates face-to-face interventions with web-based technologies. This interpretation is in line with the notion that more severely depressed patients see the availability of an online program between face-to-face sessions as an advantage of the blended treatment [85]. Furthermore, healthcare utilization should be evaluated prior to treatment selection, because it can give valuable information about the patients’ needs, treatment history, and course of illness. In addition, the predictors found to be important in this study and in previous studies could be taken into account to make an informed treatment recommendation in clinical practice. However, future studies with larger samples and more advanced techniques are necessary to validate the current findings, and the identified predictors have to be tested within clinical routine treatment settings. Moreover, prospective studies need to integrate the PAI in the diagnostics process at the beginning of a treatment.

## 5. Conclusions

To conclude, with the first aim of the study, two prognostic predictors, namely, pretreatment symptomatology and treatment expectancy, were found. Furthermore, several prescriptive predictors were found, predicting the treatment outcome respective of each of the two conditions. Some of our findings are in line with previous research, but other variables, such as baseline healthcare utilization, have not been investigated in this context. The interpretations regarding the prognostic and prescriptive predictors need to be tested empirically, because they are somewhat speculative. Furthermore, this study showed an advantage of model-determined treatment allocation to the TAU or blended treatment, as one-third of the participants had a PAI larger than 5, which means they would have improved significantly if they had received their “optimal” treatment. Although the results need to be validated in future hypothesis-driven studies, the predictors found to be important in the current study should be taken into account to make an informed treatment recommendation in clinical practice.

## Figures and Tables

**Table 1:** TiC-P items included in the analysis.

“How many times did you consult a general practitioner?” “How many times did you consult a psychologist?” “How many times did you consult a psychotherapist?”, “How many times did you consult a psychiatrist?”, and “How many times did you consult a professional from an ambulatory mental health institution?” “How many times did you consult a professional from a clinic for alcohol or drugs?” and “How many times did you consult self-help groups?”	“How many days did you spend in a day-time treatment program in a regular hospital?” “How many days did you spend in a day-time treatment program in a psychiatric hospital?” and “How many days did you use outpatient psychotherapeutic services in addition to your psychotherapy?”	“How many admissions to a regular hospital did you have?” and “How many admissions to a psychiatric hospital did you have?” “How many admissions to a rehabilitation clinic did you have?” “Do you have a paid job?” “Did health problems oblige you to call in sick from work at any time?”

**Table 2:** Five best models for treatment as usual (TAU).

Fit Indices	Model 1	Model 2	Model 3	Model 4	Model 5
Bayes Factor	1	0.934	0.597	0.572	0.510
Number of Variables	6	6	6	5	6
R^2^	0.428	0.445	0.441	0.440	0.439
Log Marginal Likelihood	22.729	22.659	22.213	22.170	22.056
Posterior Probabilities	0.021	0.019	0.012	0.012	0.011

**Table 3:** Five best models for the blended treatment condition.

Fit Indices	Model 1	Model 2	Model 3	Model 4	Model 5
Bayes Factor	1	0.887	0.577	0.525	0.516
Number of Variables	6	5	4	6	6
R^2^	0.398	0.416	0.392	0.429	0.446
Log Marginal Likelihood	19.852	19.731	19.301	19.207	19.190
Posterior Probabilities	0.012	0.011	0.007	0.006	0.006

## Supplementary Materials

The following are available online at https://www.mdpi.com/2077-0383/9/2/490/s1: Appendix 1: Bayesian model averaging (BMA) results based on the best 30,000 models in the treatment as usual (TAU) condition, Appendix 2: BMA results based on the best 30,000 models in the blended treatment condition.
