# Supplementary material for: Using the Personalized Advantage Index for Individual Treatment Allocation to Blended Treatment or Treatment as Usual for Depression in Secondary Care
Source: J Clin Med. 2020 Feb 11;9(2):490. doi: 10.3390/jcm9020490 (PMC7073663; doi:10.3390/jcm9020490)
Supplement: Supplementary file 1 [file jcm-09-00490-s001.pdf]

Appendices

Appendix 1

BMA results based on the best 30000 models in the TAU condition

Baseline variables	Prob (%)	Posterior mean (SD)
Sociodemographic variables		
Denmark	57.5	-1.572179 (1.682192)
High education	33.4	-0.498814 (0.890317)
Middle education	15.4	0.152283 (0.525018)
Married	14.4	-0.164144 (0.532724)
Age	8.5	-0.001673 (0.010853)
Divorced	7.7	0.076725 (0.481731)
Female gender	6.1	-0.046692 (0.289896)
Switzerland	6.1	-0.041385 (0.365273)
France	4.6	-0.018721 (0.245178)
Living together	4.5	-0.006237 (0.229171)
Widowed	3.7	-0.007041 (0.911245)
Symptomatology and quality of life		
pre-treatment PHQ-9	100	0.568373 (0.090957)
Therapy preference blended treatment	14.3	0.150752 (0.513971)
Therapy preference TAU	7.9	-0.051866 (0.329026)
Dysthymia	7.7	-0.090115 (0.555429)
Melancholic	7.2	-0.020811 (0.256394)
Recurrent depression	7.2	-0.041963 (0.275208)
Anxiety disorder	7	0.045272 (0.281162)
Antidepressant medication	6.6	-0.033555 (0.262399)
EQ-5D	3.4	0.019908 (0.404468)
Healthcare utilization		
"How many days did you use outpatient psychotherapeutic services in addition to your psychotherapy?"	94.5	-8.179901 (3.765576)
"How many times did you consult a general practitioner?"	63.5	-0.043966 (0.120152)

"How many days did you spend in a day-time treatment program in a psychiatric hospital?"	50.5	3.533107 (4.372049)
"How many times did you consult a professional from a clinic for alcohol or drugs?"	37.3	-5.172186 (8.222200)
"How many times did you consult a psychologist?"	30.4	0.083479 (0.163982)
"How many times did you consult a professional from an ambulatory mental health institution?"	22.1	-0.044135 (0.108400)
"How many times did you consult a general practitioner?"	18.8	-0.043966 (0.120152)
"How many times did you consult self-help groups?"	14.1	-0.548520 (1.867824)
"Did health problems oblige you to call in sick from work at any time?"	13.1	0.156083 (0.592364)
"How many admissions to a psychiatric hospital did you have?"	8.5	-0.159826 (0.882893)
"Do you have a paid job?"	7.8	-0.055734 (0.313400)
"How many admissions to a regular hospital did you have?"	6.8	-0.059203 (0.489230)
"How many days did you spend in a day-time treatment program in a regular hospital?"	6.3	0.089278 (0.858992)
"How many times did you consult a psychotherapist?"	5.8	-0.001238 (0.043720)
Patient expectancy		
CEQ expectancy	97.5	-0.267220 (0.106232)
CEQ credibility	7	0.003196 (0.032870)

Appendix 2

BMA results based on the best 30000 models in the blended treatment condition

Baseline variables	Prob (%)	Posterior mean (SD)
Sociodemographic variables		
Widowed	49.7	4.251648 (5.427225)
Switzerland	35	-0.743754 (1.257351
Married	32.2	-0.483091 (0.883834)
Middle education	31.7	0.437548 (0.820269)
Divorced	15.6	0.220980 (0.720755)
High education	12	-0.112651 (0.486546)
Denmark	10.1	-0.114511 (0.525979)
Age	8.7	-0.002926 (0.014872)
France	8.4	0.065530 (0.384302)
Living together	7.6	0.054765 (0.374650)
Female gender	5.1	0.024376)
Symptomatology and quality of life		

pre-treatment PHQ-9	100	0.368370 (0.109324)
EQ-5D	74.6	-3.670476 (2.893040)
Dysthymia	32.4	-0.996616 (1.809627)
Melancholic	9.6	0.091906 (0.443747)
Therapy preference TAU	6.9	0.018880 (0.281612)
Anxiety disorder	5.5	-0.996616 (1.809627)
Recurrent depression	4.8	-0.008765 (0.197716)
Therapy preference blended treatment	4.8	-0.013855 (0.231330)
Antidepressant medication	4.7	-0.007456 (0.208791)
Healthcare utilization		
"How many admissions to a regular hospital did you have?"	99.9	8.342981 (2.151191)
"How many times did you consult self-help groups?"	70	7.127134 (6.193637)
"How many times did you consult a professional from an ambulatory mental health institution?"	12.99	-0.022874 (0.090434)
"How many times did you consult a psychiatrist?"	12.9	-0.028600 (0.114615)
"How many times did you consult a general practitioner?"	7.7	-0.011800 (0.065338)
"Did health problems oblige you to call in sick from work at any time?"	6.9	0.047023 (0.361087)
"How many times did you consult a professional from a clinic for alcohol or drugs?"	6.8	0.110948 (0.805541)
"How many times did you consult a psychologist?"	6.5	-0.005287 (0.054229)
"How many days did you spend in a day-time treatment program in a regular hospital?"	6.4	0.139228 (1.344264)
"How many days did you spend in a day-time treatment program in a psychiatric hospital?"	5.9	0.029929 (0.701060)
"How many admissions to a psychiatric hospital did you have?"	5.7	-0.006769 (0.843517)
"How many times did you consult a psychotherapist?"	5	0.005185 (0.053066)
How many days did you use outpatient psychotherapeutic services in addition to your psychotherapy?"	4.9	0.008775 (0.774172)
"Do you have a paid job?"	4.9	-0.003680 (0.206993)
Patient expectancy		
CEQ expectancy	72.4	-0.221924 (0.170339)
CEQ credibility	43.9	-0.128043 (0.175279)
